# A Battery of Jump Tests Helps Discriminating Between Subjects With and Without Chronic Ankle Instability

**DOI:** 10.3390/sports13060171

**Published:** 2025-05-30

**Authors:** Claudio Legnani, Matteo Saladini, Martina Faraldi, Giuseppe M. Peretti, Alberto Ventura

**Affiliations:** 1 Sport Traumatology and Minimally Invasive Surgery Center, IRCCS Istituto Ortopedico Galeazzi, 20148 Milan, Italy; 2 Residency Program in Orthopedics and Traumatology, University of Milan, 20122 Milan, Italy; 3 Laboratory of Experimental Biochemistry and Advanced Diagnostics, IRCCS Istituto Ortopedico Galeazzi, 20157 Milan, Italy; 4 E.U.O.R.R. Unit, IRCCS Istituto Ortopedico Galeazzi, 20157 Milan, Italy; 5 Department of Biomedical Sciences for Health, University of Milan, 20122 Milan, Italy

**Keywords:** ankle, chronic ankle instability, vertical jump, battery test

## Abstract

The purpose of this study was to assess whether a simple and reproducible battery of jump tests can distinguish between patients affected by chronic ankle instability (CAI) and control subjects. The hypothesis was that patients with CAI would demonstrate lower performance compared to healthy subjects during jumping tasks. Twenty-one young, active adults aged 18 to 45 years affected by CAI were matched for sex, age, and body mass index (BMI) to a control group of 21 healthy subjects without history of lower limb pathology. Jumping ability was instrumentally assessed by an infrared optical acquisition system using a test battery, including mono- and bipodalic vertical squat jumps, countermovement jumps (CMJs), a drop jump (DJ), and a side-hop test. Patients with CAI had significantly worse monopodalic CMJ, DJ, and side-hop test scores in their involved limb compared to the non-dominant limb of healthy individuals. Pathological limbs of CAI patients reported inferior results compared to non-dominant limbs of healthy individuals while performing monopodalic CMJs, DJs, and side-hop tests (*p* < 0.05). No statistically significant differences were found between the two groups in the limb symmetry index (LSI) while performing monopodalic CMJs and DJs (*p* = 0.072 and *p* = 0.071, respectively), while a difference was found between the two groups, in favor of healthy subjects, while performing monopodalic side-hop tests (*p* < 0.01). A reproducible battery of jump tests performed with a simple and low-cost instrument can be applied in the clinical setting allowing for reliable measurements of functional ability of subjects with CAI. Our findings support the idea that side-hop tests could be more accurate than vertical jump tests for detecting functional deficits in patients suffering from CAI.

## 1. Introduction

Sports involving jumping and landing, such as basketball, volleyball, and soccer, have been known to frequently cause ankle sprains. These injuries, if left untreated, can lead to a condition of joint disability called chronic ankle instability (CAI) [1]. CAI is a condition characterized by recurrent “giving way” episodes, not infrequently associated with pain and reduced joint function, which stem from damage to the lateral ligaments of the ankle. Previous studies demonstrated that patients with CAI have altered strength, neuromuscular function, balance and postural control, ground reaction force, and gait when compared to healthy persons [2,3]. Evidence shows that jump battery tests, which measure knee extensor strength, explosive power, and coordination, can investigate the biomechanics of the lower limbs [4,5]. Assessing patients with pathologic knee diseases, such as those following anterior cruciate ligament reconstruction, has been made easier with the help of these tasks [6].

Jumping ability is an important indicator of athleticism in patients performing cutting and pivoting sports; its evaluation demonstrated to be a reliable and sensitive test for determining deficits related to pathology [7]. Therefore, deficiencies in neuromuscular function when performing tasks involving jumping and landing during sports participation could indicate a malfunctioning joint-protecting control mechanism when the joint is positioned harmfully during locomotion [8]. The biomechanics of bipodalic and monopodalic vertical jump and jump-landing tasks in patients with CAI have not been extensively studied. There have been reports of neuromuscular alterations during strength and balance tests, as well as while walking [9,10,11]. Although jump battery tests are frequently employed in research, there are few clinical uses for them, and their ability to detect deficits related to pathology is still debated.

Previous studies for identifying functional limitations related to CAI have only included the use of one functional test in order to assess lower limb biomechanics, but to evaluate differences in functional capacities, multiple dynamic tests should be used to evaluate joint kinematics. The use of a battery encompassing different jump tests may better investigate different aspects of the athletic gesture: explosive strength, balance, and neuromuscular control. Such assessments may provide more solid and reliable data about differences between patients affected by CAI and healthy participants.

The purpose of this study was to assess whether a jump battery test can distinguish between patients affected by CAI and control subjects. The hypothesis was that patients with CAI would demonstrate lower performance compared to healthy subjects during jumping tasks and that the amount of impairment may differ depending on the test performed.

## 2. Materials and Methods

### 2.1. Patients Recruitment

Twenty-five patients with CAI were recruited for the present study at the Sport Traumatology and Minimally Invasive Surgery Center of the Galeazzi Orthopedic Institute. CAI was defined according to the standard criteria endorsed by the International Ankle Consortium [12].

Four patients of the study group were screened out because they did not meet inclusion criteria. The remaining 21 patients were matched for age, male:female ratio, and BMI to a control group of 21 healthy subjects with no history of ankle sprains. IRCCS San Raffaele Hospital’s Ethics Committee, Milan, Italy, approved this study (IRB number: 177/INT/2022). All patients signed informed consent.

Inclusion criteria for both groups were as follows: age 18–45 years, BMI (kg/m^2^) ≤ 30, absence of neurological disorders (e.g., epilepsy, stroke) or any condition impairing the ability to perform jumping assessment. Patients were excluded if they had previous ankle surgery (including the contralateral limb), refused to sign the consent form, or for females if they were pregnant at the time of evaluation.

### 2.2. Patients’ Assessment

A series of jump tests was executed in accordance with a previous protocol [13], using an infrared optical acquisition system (OptoGait; Microgate, Bolzano, Italy), composed by a transmitting and a receiving photoelectric bar. Bars were placed at a distance of 2.5 m from each other at the ground surface level. Patients were instructed to warm up 10 min while performing practice submaximal jump trials. The test battery included bipodalic squat jump (SJ), bipodalic countermovement jump (CMJ), monopodalic CMJ, monopodalic drop jump (DJ), and monopodalic side-hop test performed with the uninjured limb first, followed by the injured. Each functional test was executed three times with the exception of the DJ and the side-hop test, which were performed once for each limb. Test–retest reliability of these jump tests was good to excellent, as previously reported, with intraclass correlation coefficients ranging from 0.72 to 0.97 [14]. Data collection was performed by two experienced researchers.

During the SJ, participants jumped vertically, starting from a semi-squatting position without countermovement. When performing the CMJ, participants started from the upright position and performed a quick squat followed by a maximal vertical jump. During jumping, the subjects were required to keep their hands on their hips and land with straight legs. The DJ was performed with the subject standing on a 30 cm box with a target line drawn 40 cm in front of the box. The subjects jumped down on one leg, landing just past the marked line, and immediately performed a one-legged maximum vertical jump. Vertical height was recorded. The side-hop test required subjects to perform as many as possible lateral monopodalic lateral jumps over 2 parallel strips placed 30 cm apart, within 30 s, and the number of jumps in this time frame was recorded. When performing monopodalic jumps, first the non-injured/dominant side was tested, followed by the injured/non-dominant side for each participant. Limb dominance was determined by asking which leg subjects thought was their dominant leg (e.g., the leg with which they would preferably kick a ball).

Appropriate resting time (3 to 5 min) was allowed between each jumping performance to avoid fatigue. Test scores were recorded as flight time (in milliseconds) and distance (in centimeters) using Optogait PC Software Version 1.12.0 and calculated as the mean of the trials performed. The limb symmetry index (LSI) was calculated from the following formula:

$$LSI=\frac{Value\;of\;the\;injured/non-dominant\;limb}{Value\;of\;the\;uninjured/dominant\;limb}\times 100$$

### 2.3. Statistical Analysis

Data were analyzed using Graphpad Prism v8.0 (Prism Software, La Jolla, CA, USA). Categorical variables were reported as absolute and relative frequencies for each group, and comparisons were performed using Fisher’s exact test. The normal distribution of continuous variables was analyzed using the Shapiro–Wilk test. Data were compared using the Mann–Whitney test or multiple comparisons with the Holm–Sidack correction. The sample size was calculated considering the difference in the monopodalic CMJ between the two groups as the primary outcome. Considering the monopodalic CMJ mean and SD in the control group of 38.1 ± 6.7 cm based on a previous study [15], hypothesizing a between-groups difference of 5 cm, setting the significance and power at 0.05 and 80%, respectively, 42 participants were required, 21 for each group.

## 3. Results

Patients’ demographics and anthropometrics were not significantly different between the two groups (Table 1).

Of the 21 patients affected by CAI, 12 had right ankle and nine had left ankle instability. The injured limbs were the dominant limb in 12 patients (57%). In the control group, the dominant limb was right in 17 of 21 subjects (81%). Significant differences (lower in CAI patients compared to healthy individuals) have been observed for the monopodalic CMJ, DJ, and side-hop tests between non-dominant limbs of healthy individuals compared to pathological limbs of CAI patients (*p* < 0.05, Table 2).

**Figure 1:**
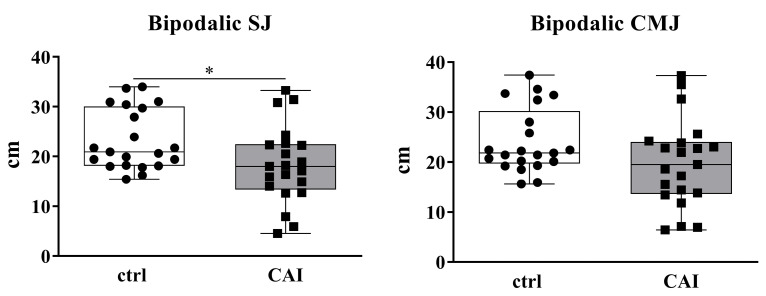
Bipodalic squat jump (SJ) and countermovement jump (CMJ). Comparisons between patients with chronic ankle instability (CAI) and healthy individuals (ctrl) have been performed using Mann–Whitney test. *p*-value < 0.05 was considered significant (* *p*-value < 0.05).

The results from the bipodalic jump have shown a significant decrease in bipodalic SJs in CAI patients compared to healthy individuals (*p*-value = 0.036) and no statistically significant differences in bipodalic CMJs between healthy individuals and CAI patients (*p*-value = 0.144) (Figure 1).

**Figure 2:**
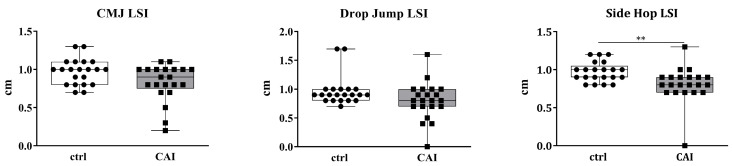
Countermovement jump (CMJ), monopodalic CMJ, monopodalic drop jump (DJ), monopodalic countermovement jump (CMJ), drop jump (DJ), and side-hop limb symmetry index (LSI) between-group analysis. Comparisons between patients with chronic ankle instability (CAI) and healthy individuals (ctrl) have been performed using Mann–Whitney test. *p*-value < 0.05 was considered significant (** *p*-value < 0.01;).

Although no statistically significant differences were found, a consistent reduction in CMJ and DJ LSI was observed in patients with CAI compared to healthy individuals (*p* = 0.072, *p* = 0.071, respectively; Table 3, Figure 2). A statistically significant difference was found between the two groups, in favor of healthy subjects, in the LSI while performing the monopodalic side-hop test (*p* < 0.01, Table 3, Figure 1). Concerning the side-hop test, the mean number of side-hop was seven repetitions (16%) fewer for the injured side compared to the non-injured side in patients with CAI (*p* < 0.001), while no difference was noted among healthy individuals (*p* = n.s.).

## 4. Discussion

According to our findings, the pathological limb of patients with CAI had significantly lower results compared to the non-dominant limb of healthy individuals while performing monopodalic CMJs, DJs, and side-hop tests. There was also a significant difference in LSI between patients affected by CAI and the control group while performing the side-hop test, but not while executing CMJs and DJs.

CAI is associated with changes in kinematic patterns during gait and sporting activities, and functional test batteries have been designed in addition to patient-reported outcome measures to investigate impairments in patients with CAI aiming to lead an active lifestyle [16]. According to previous studies, the vertical jump can reliably measure quadriceps explosive power, strength, and lower limb neuromuscular control [17,18,19,20]. In the present study, a series of vertical and lateral jump tests was used to detect asymmetries between limbs and between patients with CAI and healthy subjects, thus determining the level of impairment affecting patients with CAI.

In the current study, the jumping ability of the injured side in the patients affected by CAI was significantly lower compared to the uninjured side while performing the side-hop test, as demonstrated by the LSI. Although a trend for lower performance was reported also for CMJs and DJs, our study failed to detect statistical significance. Similarly, Augustsson and Sjöstedt observed that patients affected by CAI who have the greatest impairments in side-hop ability and balance appear to have deficiencies in muscle strength, balance, and functional performance [21]. A little divergence from the outcome revealed in our study was observed in another study, which found that CAI patients performed 20% better on the non-damaged ankle compared to the affected side in a single-leg balance test [22]. Nonetheless, different testing techniques were applied to assess balance, which might have affected the outcomes. Reduced jump performance while performing side-hop tests may provide insight as to why individuals experiencing CAI exhibit decreased lower limb function; in fact, lateral jump landings are responsible for increased stress on the lateral joint structures, thus affecting eversion muscles and influencing jumping ability [23]. In patients suffering from CAI, functional deficits may lead to abnormalities in ankle joint function, which could affect movement strategies during side-cutting tasks.

Conversely, no significant differences have been found in LSI for monopodalic CMJs and DJs in patients affected by CAI. These findings suggest that these tests may be less accurate for measuring explosive strength and balance in CAI patients. In fact, by loading the foot sagittally, CMJ and DJ tests may not be as challenging for the lateral ligamental joint complex as the side-hop test in patients suffering from CAI [21]. Similarly, Hiller et al. could not detect significant variations in ankle balance between injured and uninjured ankles. Rather, it was discovered that individuals with unilateral injuries had impairments on both sides. Nonetheless, there was a two-fold increase in the rate of foot lifts in the wounded group as compared to the controls and those who had instability [24]. On the other hand, previous studies have shown that participants with and without ankle instability exhibit different landing patterns and force distributions during jump landings [25].

Several studies have been conducted to detect impairments in the functional performance of patients with CAI compared to healthy subjects, as well as to “copers” [26]. Jump battery tests are frequently employed in research for their ability to detect deficits related to pathology. However, a significant obstacle to the application of these potentially helpful tests in clinical practice has been the wide variations in methodology, end measures, and findings, as well as the use of sophisticated tools like force plates and isokinetic dynamometers. To assess the functional abilities of patients with CAI, clinical practitioners would benefit from the development of readily repeatable functional performance tests backed by reasonably priced and conveniently available technologies. Furthermore, standardized assessments that can be compared between patients and at various intervals during a patient’s rehabilitation may be made possible by these straightforward outcome measuring techniques.

Eventually, understanding which functional tests have the higher potential to detect impairments in subjects suffering from CAI could help to set realistic rehabilitation goals and predict return-to-sports timing.

Limitations of the present study include the relatively small sample size, which makes it more difficult to identify subtle variations between groups concerning certain variables. OptoGait was selected as a straightforward, affordable tool that is easy to utilize in the clinical setting and allows for reliable measurements of functional ability. Its validity concerning the evaluation of spatiotemporal gait parameters has been previously reported [27]. Many variables influence jumping performance, and the use of jumping capacity as an expression of neuromuscular performance to investigate ankle pathology constitutes a further study limitation. In addition, normalizing jump test results to anthropometric parameters would have increased the accuracy of results.

Future studies with larger cohorts and different tests are required to investigate the correlation between the variables affecting ankle performance to improve rehabilitation strategies for the treatment of ankle joint pathology.

## 5. Conclusions

The pathological limb of CAI patients reported inferior results compared to the non-dominant limb of healthy individuals while performing monopodalic CMJs, DJs, and side-hop tests. Concerning between-limbs results, LSI in the patients affected by CAI was significantly lower compared to the uninjured side while performing the side-hop test, but not while executing CMJs and DJs. These findings suggest that the side-hop test could be more accurate than vertical jump tests for detecting functional deficits in patients suffering from CAI.

## Figures and Tables

**Table 1:** Patient demographics and anthropometric data. M:F ratio has been analyzed using Fisher’s exact test; age and BMI have been analyzed using Mann–Whitney test. *p*-value < 0.05 was considered significant.

	CAI Patients	Control Group	*p*-Value
No. of patients	21	21	
M:F ratio	16:5	16:5	>0.999
Mean age at surgery ± SD (yr)	33.1 ± 8.2	32.8 ± 7.6	0.945
Mean BMI ± SD (kg/m^2^)	23.0 ± 2.3	23.3 ± 2.7	0.916

CAI: chronic ankle instability; SD: standard deviation; BMI: body mass index.

**Table 2:** Monopodalic CMJ, DJ, and side-hop analysis in patients with chronic ankle instability (CAI patients) and healthy individuals (control group). Data are expressed as mean ± SD and median and interquartile range (IQR). Within-group and between-group comparisons were performed by multiple comparisons with Holm–Sidack correction. In bold are reported significant comparisons (*p*-value < 0.05).

	CAI Patients (n = 21)			Control Group (n = 21)				
	Uninjured Limb	Pathological Limb	*p*-Value	Dominant Limb	Non-Dominant Limb	*p*-Value	*p*-Value of Dominant Limb Control Group vs. Uninjured Limb CAI	*p*-Value of Non-Dominant Limb Control Group vs. Pathological Limb CAI
Monopodalic CMJ (cm)	9.59 ± 4.12 9.60 (7.00–12.30)	8.31 ± 4.18 9.20 (5.40–10.40)	0.327	12.50 ± 3.83 12.00 (10.05–16.05)	11.95 ± 3.80 10.50 (8.65–15.80)	0.644	**0.023**	**0.005**
Monopodalic DJ (cm)	11.08 ± 5.91 10.70 (8.75–14.95)	9.28 ± 5.74 8.10 (4.35–13.60)	0.157	13.86 ± 5.02 12.70 (9.70–17.95)	12.90 ± 4.18 10.80 (10.20–16.30)	0.504	0.247	**0.025**
Monopodalic side-hop (cm)	43.90 ± 17.30 41.00 (30.00–58.50)	37.19 ± 19.37 33.00 (24.00–55.00)	0.243	51.00 ± 18.01 49.00 (40.00–56.00)	49.71 ± 19.30 43.00 (38.50–59.50)	0.824	0.200	**0.042**

CAI: chronic ankle instability; CMJ: countermovement jump; DJ: drop jump.

**Table 3:** Monopodalic CMJ, DJ, and side-hop between-group analysis. Data are expressed as mean ± SD and median (IQR). Comparison between patients with chronic ankle instability (CAI patients) and healthy individuals (control group) have been performed using Mann–Whitney test. In bold are reported significant comparisons (*p*-value < 0.05).

	CAI Patients (n = 21)	Control Group (n = 21)	*p*-Value
CMJ LSI	0.83 ± 0.24 0.90 (0.75–1.00)	0.97 ± 0.17 1.00 (0.80–1.10)	0.072
DJ LSI	0.80 ± 0.32 0.80 (0.70–1.00)	0.97 ± 0.26 0.90 (0.80–1.00)	0.071
Side-hop LSI	0.81 ± 0.23 0.80 (0.70–0.90)	0.97 ± 0.13 1.00 (0.90–1.05)	**0.002**

CAI: chronic ankle instability; SD: standard deviation; IQR: interquartile range; CMJ: countermovement jump; DJ: drop jump; LSI: limb symmetry index.

## Data Availability

Raw data are available at the following link: https://zenodo.org/records/15496221 (accessed on 23 May 2025).
